# Editorial for the Special Issue on Optical Trapping and Manipulation: From Fundamentals to Applications

**DOI:** 10.3390/mi11040417

**Published:** 2020-04-15

**Authors:** Daniel R. Burnham, Philip H. Jones

**Affiliations:** 1 The Francis Crick Institute, 1 Midland Road, London NW1 1AT, UK; 2 Department of Physics & Astronomy, University College London, Gower Street, London WC1E 6BT, UK

This Special Issue of *Micromachines* is devoted to optical trapping, and the enormous range of uses the method has found in the decades since its first demonstration. The papers published here include both novel research articles and in-depth reviews, innovative optical trapping schemes and notable new applications. This diversity of the research field is reflected in the breadth of papers contained in this Special Issue. 

Two papers are concerned with the investigation of mechanical interactions of red blood cells (RBCs). Zhu et al. have investigated the disaggregation of RBCs by using optical tweezers to quantify the force required to separate aggregated cells, showing that a short irradiation from a pulsed helium-neon laser may reduce the aggregation force [1]. Avsievich et al. report on the effect of polymeric nanocapsules—a model for drug delivery vehicles—on the aggregation force of RBCs, finding no change and no cytotoxicity as a result of the nanocapsule treatment [2].

Vivek et al. have used optical tweezers to study biomembranes, by inducing the controlled fusion of giant unilamellar vesicles (GUVs) [3]. The manipulation of membrane components of such ‘artificial cells’ provides great insight into the fusion process and morphological transitions that occur during fusion, and the authors exploit the spatial and temporal control provided by optical trapping to observe the fine details of fusion events.

Optical tweezers also find application in single-molecule biophysics, such as the work reported by Kretzer et al. [4] By combining optical tweezers into a microfluidic device, the authors were able to measure the concentration-dependent effects on the mechanics of single molecule ligand binding.

A novel optical trapping scheme utilizing a micro-ring resonator is reported by Ho et al., who used the evanescent field of the device to trap microscopic particles [5]. The system uses the resonant enhancement of the field in the resonator, together with a closed-loop system that renders the trapping insensitive to changes in the environment.

In their communication, Shishkin et al. reported on the development of microscopic optomechanical tools for use with optical tweezers [6]. They demonstrate the fabrication of a clamping fork that can be used to hold cells and is capable or arbitrary three-dimensional manipulation and also rotations. Such microtools are advantageous for enhancing the range of manipulations that may be applied to trapped objects.

Microscopic optically trapped and actuated machines are considered in depth in the review article by Andrew et al. [7]. The authors give a motivation for the use of such microrobots for avoiding optical damage to delicate biological specimens, and present the evolution of a number of different designs for a range of applications.

Finally, Zhao et al. have provided an in-depth review of an alternative optical trapping scheme, namely optical fibre tweezers [8]. They have provided a comprehensive overview of a number of different optical trapping configurations that use optical fibres, including dual fibre, single fibre and structured fibre traps; the benefits such schemes may have over conventional optical tweezers, and the range of applications they have found.

We would like to thank all the authors who have contributed their work to this Special Issue, and all the referees who have dedicated their time to the rigorous reviewing process.

In closing this editorial, we would like to remind readers that a second volume of *Optical Trapping and Manipulation: from Fundamentals to Applications* is accepting contributions until 31 January 2021, for publication later that year.
